# Selections

**Published:** 1859-07

**Authors:** 


### Selections.

☞THE following extracts, from a lecture, by Dr. B. W. RICHARDSON, delivered before the College of Dentists of England, treats concisely of agents that exert a pernicious influence upon the teeth, both direct and indirect. He takes each agent separately, and briefly sets it up in full view, so that it may be examined in detail. An analytical investigation of the various causes that produce disease of the teeth, has received comparatively but little attention, especially by dentists of our own country. We have busied ourselves with the art, to the neglect of the science, of the profession. The declaration that has been so frequently made, that the Profession in the United States is in advance of that of any other country, is only true, if at all, in the practical department. These lectures of Dr. R.'s have an analytical character which we are much pleased to see. There is a deep hold taken upon the subjects under discussion, and not a skimming-over the surface, as we too frequently see. These lectures are valuable to all, but especially to the dental student.—Ed.

But one poison, much more important than any other to the surgeon-dentist, is of the class that passes through the system to the tooth-structure, and inflicts its mischief in no other way. I refer to *mercury*. We have experimental proof of the constitutional medium in this case. The poison may be rubbed into a limb, may be taken by the mouth, may be inhaled in fumes, but the effect, if the effect is carried far enough, will, *cæteris paribus*, be the same as regards the dental structures. The partially disintegrated and poisoned blood, as it bathes the tissues of the body, injects the vascular structures and refuses nutrition to those of fibrous or vascular build; hence the mercurial malady in relation to the teeth begins not in the tooth structure truly, not in the surrounding bone structure truly, but in the membrane that connects tooth and bone—the periosteum, and in the vascular structure which encircles the teeth to their crowns—the gum. In the after steps of the process, the body of the tooth does, however, in its turn suffer; partly, it may be, from the tooth itself being bathed in poisoned saliva, but more decidedly from the destruction of the connection between the tooth itself and its socket, and, by implication, of the vessels which supply to the inner structure of the teeth their nutritive pabulum.

It is a matter as yet open for inquiry, how far some other common poisons introduced into the body may exert an influence on the nutrition of the tooth. I have heard of *iodine* producing injurious results this way, but can find no reliable data of the fact; and in such cases as the iodism produced by iodine have been observed by myself (to the best of my recollection in three cases), no injurious results to the teeth followed.

I saw a case once in which *oxalic acid* was taken in a poisonous quantity, but in which the patient recovered: in this case profuse salivation was a leading symptom, and at one time every tooth was loosened. During a portion of this period, as experiment proved, the saliva was taking its part in eliminating the poison, so that the teeth were constantly bathed in acidified saliva. Notwithstanding this, as recovery progressed, the teeth became again firm in their sockets, and appeared, when convalescence was complete, to have undergone no organic change.

The effects of *arsenic* taken in gradual doses into the system have not been, as yet, sufficiently investigated in relation to the teeth. It is known that this poison, acting through the system, produces ulceration of the mouth and of the gums, but this is all that has been ascertained.

*Antimony*, which in its systematic effects closely resembles arsenic, seems to leave both the teeth and their adjacent structures free from injury, At least, this is the result at which I have myself arrived, from observation of the effects of the poison, both in man and in inferior animals. In the course of the researches from which this deduction is drawn, eleven animals were kept under the influence of the poison for long periods.

In some examples, where antimony is carried to its extreme effects in the treatment of disease, a pustular affection extending over the whole of the body has been the result. I had the opportunity of seeing this extreme effect once produced in a case of acute pneumonia in the human subject. In this case the pustular eruption extended over the whole of the body to such extent that, in ignorance of the cause, small-pox might at first sight have been supposed present. The interior of the mouth was covered with the eruption, but the gums escaped altogether from purulent disorder, or ulceration.

The *alkalies*, when carried to excess, exert a very decided influence on the system at large, and indirectly on the teeth. The general symptoms produced by the alkalies are those of low fever, attended with fluidity of blood, convulsion, and comatose death. In their special influence on the teeth and adjacent structures, they produce sponginess of gums, disconnection of the tooth from its socket, and all the consequences, in so far as these organs are concerned, arising from the continued use of mercury. Carried far enough, all the caustic alkalies, viz: potash, soda, and ammonia, appear to have the same effect on the inferior animals; and it is interesting to know that a similar effect may be produced by them in man.

I have seen potash water administered to such persistent effect, that a state resembling ptyalism was the result, but the most notable illustration of the effect of alkaline bodies is the following case, recorded by the famous Dr. Huxham:

"I had once," says Dr. Huxham, "under my care a gentleman of fortune and family, who so habituated himself to the use of vast quantities of the volatile salt that ladies commonly smell to—carbonate of ammonia—that at length he would eat it, in a very astonishing manner, as other people eat sugared carroway-seeds, *with a vengeance.* The consequence was that he brought on a hectic fever, vast hæmorrhages from the intestines, nose, and gums, while every one of his teeth dropped out, and he could eat nothing solid. He wasted vastly in flesh, and his muscles became as soft and flabby as those of a new-born infant. He broke out all over his body in pustules. He was at last with great difficulty persuaded to leave off this pernicious custom. He rubbed on in a miserable manner for several months, but died tabid and and in the highest degree of a marasmus."

The constitutional effects produced by the long-continued use of *opium* remain to be more carefully studied in reference to its action as a poison on the masticatory organs. In confirmed opium eaters, the gums in which the teeth are firmly set have a shrunken and pale character: the teeth themselves presenting little indication of disease. This observation relates to adults who are given to the practice of taking opium in large quantities, and one might infer from this that opium does not affect specially the dental structures; but I am inclined to think that among the children of the poor who are drugged with opium, not only is the second dentition seriously impeded, but the temporary teeth are small and dark, deficient in enamel, and very liable to caries.

To sum up—

The following are the propositions which I would respectfully submit as expressing in a few words the relationships which exist between constitutional causes and dental diseases:

1. Dental diseases of whatever kind have two sources; a simple local source, and a constitutional source.

2. The diseases of the organs of mastication, arising purely from local causes, are limited in number; including caries, necrosis, inflammation or its upshot, and the ache, consequent upon exposure of nerve.

3. Every form of tooth disease may directly or indirectly originate in constitutional disorder, and some originate in no other ways.

4. In all such cases the true cause of the disease is external, and the system is merely the medium by which the cause passes, or through which it is carried to the locally diseased structure.

5. The causes acting on the teeth to produce diseases through the system, are of two kinds—chemical, as by introduction of poison into the body—and physiological, as by perversion of normal acts.

6. The inorganic poisons which through the system seem mostly to affect the teeth and adjacent parts, are the mercury, the alkalies, and, perhaps, the malaria; the organic are the poisons of syphilis and small-pox.

7. *Mercury* produces caries, suppuartion, and hæmorrhage from the gums; *syphilis*, atrophy, and tendency to caries; *small-pox*, suppuration, alveolar exfoliation and dental necrosis, and *malarial poison* intermittent tooth neuralgia.

8. Among the diatheses or dispositions of body affecting the teeth, the strumous, the dyspeptic, the gouty, the rheumatic, the cancerous, and the hæmorrhagic, are the most important.

9. The *strumous* diathesis, while it favors the development of all diseases of degenerative type, is not *per se*—*i. e.*, like syphilis or small-pox—a constitutional cause of dental disease. *Simple dyspepsia* is not a true constitutional cause. *Gout* is a true constitutional cause, leading both to acute ache and organic mischief; and *rheumatism* is a constitutional cause, giving rise to a specific rheumatic neuralgia of the tooth nerve and to a specific periostitis.

That there may be other forms of constitutional disorder arising from external causes, by which dental diseases are originated and intensified, is possible and probable. But modern science, carefully read, yields at present no more than I have in adequately marked out, except one, which has been intentionally omitted.

The *hæmorrhagic diathesis* as a constitutional malady, comes strictly under our present head. But this condition being one of peculiar import, must, as the novelists have it, be considered in a chapter of its own.

